# An Evolutionary Perspective on Epistasis and the Missing Heritability

**DOI:** 10.1371/journal.pgen.1003295

**Published:** 2013-02-28

**Authors:** Gibran Hemani, Sara Knott, Chris Haley

**Affiliations:** 1 The Roslin Institute and Royal (Dick) School of Veterinary Science, University of Edinburgh, Edinburgh, United Kingdom; 2 MRC Human Genetics Unit, MRC Institute of Genetics and Molecular Medicine, University of Edinburgh, Western General Hospital, Edinburgh, United Kingdom; 3 Institute for Evolutionary Biology, University of Edinburgh, Edinburgh, United Kingdom; North Carolina State University, United States of America

## Abstract

The relative importance between additive and non-additive genetic variance has been widely argued in quantitative genetics. By approaching this question from an evolutionary perspective we show that, while additive variance can be maintained under selection at a low level for some patterns of epistasis, the majority of the genetic variance that will persist is actually non-additive. We propose that one reason that the problem of the “missing heritability” arises is because the additive genetic variation that is estimated to be contributing to the variance of a trait will most likely be an artefact of the non-additive variance that can be maintained over evolutionary time. In addition, it can be shown that even a small reduction in linkage disequilibrium between causal variants and observed SNPs rapidly erodes estimates of epistatic variance, leading to an inflation in the perceived importance of additive effects. We demonstrate that the perception of independent additive effects comprising the majority of the genetic architecture of complex traits is biased upwards and that the search for causal variants in complex traits under selection is potentially underpowered by parameterising for additive effects alone. Given dense SNP panels the detection of causal variants through genome-wide association studies may be improved by searching for epistatic effects explicitly.

## Author Summary

In this study we have shown that two independent problems may have a common cause. Why do traits under selection exhibit additive genetic variance, and why is the proportion of the heritability explained by additive effects much smaller than the total heritability estimated to exist? Our results indicate that epistatic interactions can allow deleterious mutations to persist under selection and that these interactions can abate the depletion of additive genetic variation. Furthermore, a much larger element of non-additive genetic variance is maintained, which supports the notion that the heritability estimated from family studies could be a mixture of both additive and non-additive components. We show that searching directly for epistatic effects greatly improves the discovery of variants under selection, despite the multiple testing penalty being much larger. Finally, we demonstrate that common practices in genome-wide association studies could lead to both an ascertainment bias in detecting additive effects and a confirmation bias in perceiving that most of the genetic variance is additive.

## Introduction

There exists a paradox in evolutionary biology. Despite a near-ubiquitous abundance of genetic variation [1] traits under selection often evolve more slowly than expected and, contrary to expectation, genetic variation is maintained under selection. This problem is known as ‘stasis’ [2], [3], and it is particularly evident in fitness-related traits where the genetic variation tends to be highest [4] yet there is commonly no observed response to selection at all [5]–[7]. There are a number of mechanisms by which this might arise, amongst which the most commonly cited are various forms of constraints [8], [9] or stabilising selection [10]. Because stasis is widespread its properties may reveal insights into the genetic architecture of complex traits related to fitness and thus inform the strategies that are employed to detect their underlying genetic variants. After hundreds of genome-wide association (GWA) studies [11] a picture is emerging where the total genetic variation explained by variants that have been individually mapped to complex traits is vastly lower than the amount of genetic variation expected to exist as estimated from pedigree-based studies, a phenomenon that has come to be known as the problem of the ‘missing heritability’ [12]. Again, there are probably numerous contributing factors, and ostensibly the most parsimonious explanation is that complex traits comprise many small effects that GWA studies are underpowered to detect [13], [14], but whether this is the complete story deserves exploration.

With respect to the fields of both the aforementioned issues, it is typical to model genetic variation using an additive framework, such that each allele affecting a trait acts in an independent, linear, cumulative manner. For many practical applications this is a very useful approach (*e.g.* [15], [16]), but there does exist a popular school of thought that suggests that the mechanisms of gene action, and the architecture of complex traits, are actually much more complex than the additive model allows (*e.g.* [17]–[20]). Epistasis, defined in functional terms as the event whereby the effect of one locus depends on the genotype at another locus, is one source of non-additive genetic variation. How it contributes to both the paradox of ‘stasis’ and the problem of the ‘missing heritability’ will be the focus of this study.

The importance of epistasis in complex traits has proven to be a particularly divisive issue throughout the history of quantitative genetics. Recently it has been suggested that epistasis might form part of the answer to the ‘missing heritability’ [21]–[24], but how this might manifest is not immediately obvious. When heritability estimates are reported for complex traits they typically pertain to the narrow-sense (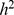, the proportion of the phenotypic variance that is due to additive genetic effects). Calculating the broad-sense heritability (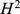, or the proportion of variance that is due to both additive and non-additive genetic effects), is an intractable problem for non-clonal populations [25], thus we have little knowledge of how much epistasis exists in human and animal traits. In this light one might suggest that we are actually dealing with two problems: the ‘missing heritability’, and the ‘unknown heritability’. By definition epistasis will form a part of the ‘unknown heritability’, but theory shows that epistatic interactions could also contribute to 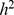 estimates. This could arise through two possible mechanisms, firstly by generating real additive variation as marginal effects from higher order genetic interactions [26]–[29]; or secondly by creating a statistical illusion of additive variance through confounding between non-additive and common environment effects in twin study based estimates [24], [30].

Beyond the realm of complex trait genetics it appears that epistasis does appear to be common. For example in molecular studies it is routine to observe ‘phenotypic rescue’ where the phenotypic effect of a gene knockout can be masked by a second knockout (*e.g.* [31]). Another commonly encountered form of epistasis is ‘canalisation’ [32], where phenotypes exhibit robustness to the knockout of one gene, requiring a second knockout to elicit a response (*e.g.* [33]). At the macroevolutionary scale, epistasis is also of central importance, for example it has recently been shown that an advantageous substitution in one species is often found to be deleterious in others, thus the substition's effect on fitness is dependent upon the genetic background in which it is found [34]. The mechanisms behind pathway-level [32], [35], [36] or species-level epistasis [20], [34], [37] are widely explored, and yet at the intermediate, within-population level there is a distinct lack of evidence for any widespread importance of epistasis arising from natural variation, and most genetic variation appears to be additive [28]. Nevertheless some convincing examples of epistasis have been reported, for instance there are a number of cases of canalisation in *Homo sapiens* [38], [39], *Gallus gallus* [40], *Drosophila melanogaster* [41], *Saccharomyces cerevisiae* [42], and *Arabidopsis thaliana* [43] to name but a few.

At the statistical level, for a pair of single nucleotide polymorphisms (SNPs) that exhibit epistasis, in addition to interaction terms between the two loci, the total genetic effect is likely to also include marginal additive or dominance effects at each locus [28], [44]. The proportion of additive to non-additive genetic variation will depend both on the genotype-phenotype map (G-P map), and the allele frequencies at each locus. In turn these frequencies will depend on selection acting on the phenotype. Thus, if epistasis contributes towards fitness then how selection acts is highly dependent on the particular genotype-phenotype map in question [45]. Ostensibly, the additive framework that is used in GWA studies follows Occam's razor, employing the hypothesis that introduces the fewest new assumptions (*i.e.* non-additive variation cannot be estimated, thus SNPs are not modelled to have non-additive effects). But whether the phenomenon of stasis can accommodate a purely additive genetic model remains an open question.

The premise of this study is centred around finding common ground between the problems of stasis and the missing heritability. Given that fitness related traits often exhibit stasis then the underlying genetic architecture may not solely comprise independent additive effects. Through theory and simulations we demonstrate that epistasis will maintain additive genetic variation under selection at higher levels than independent additive effects, and that by extending GWA studies to search for epistasis directly we could improve statistical power to detect additive genetic variation.

## Results

Selection rapidly drives deleterious additive effects to fixation, but how effective is selection at purging deleterious, non-additive effects? We simulated 56 G-P maps (including neutral, additive, dominant, and 51 epistatic two-locus patterns; Figure S1) and assuming that the phenotype had a direct effect on fitness we calculated their expected allele frequency trajectories over time. With these outcomes we were able to make inferences on i) the ability of epistasis to maintain genetic variation and the allele frequencies at which different G-P maps might stabilise, ii) the amount of genetic variation and the proportion of additive to non-additive variation that we would expect at frequencies that are evolutionarily stable, iii) the impact of incomplete linkage disequilibrium on our estimation of these G-P maps with SNP data, and iv) the relative performances of various GWA strategies at detecting additive genetic variation. The results are detailed below.

### Epistasis maintains genetic variation under selection

**Figure 1:**
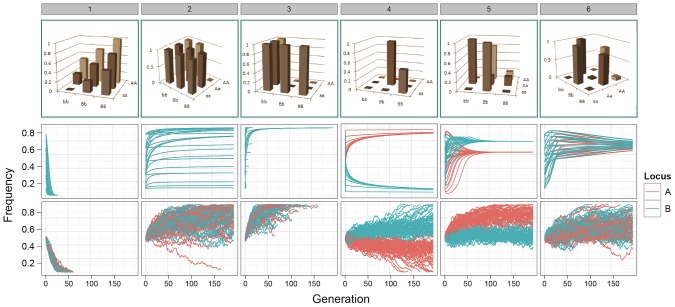
Allele frequency trajectories under selection. Top row: G-P maps. 1. Independent additive effects at locus A and B; 2. Dominant pattern of canalisation; 3. Recessive pattern of canalisation; 4–6. Patterns generated by a genetic algorithm optimising for maximised additive variance and long-term survival at intermediate frequency. Middle row: Expected allele frequency trajectories for G-P maps under selection, as derived deterministically, with initial frequencies of 0.1, 0.3, 0.5, 0.7, and 0.9 enumerated over both loci. Frequencies on the 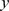-axis correspond to alleles *a/b*. Only one colour appears for patterns 1–3 because the trajectories of both alleles are identical. Bottom row: The path of allele frequencies as observed through stochastic simulations of populations comprising 1000 individuals and 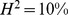 at generation 0, with initial allele frequencies at both loci of 0.5.

Our results demonstrate that for many of the patterns of epistasis that we assayed, deleterious effects can be maintained at intermediate frequencies over long evolutionary time periods (Figure 1 patterns 1–3, and Figures S1, S2 and S3). As might be expected, a number of G-P maps that maintained genetic variation at intermediate frequencies also exhibited over-dominance, or some form of heterozygote advantage (*e.g.* Figure 1 patterns 4–6). However, most patterns of epistasis that we assayed (Figure S1) do not exhibit heterozygote advantage, and these can also effectively temper the rate of extinction of deleterious alleles. Conversely, some level of under-dominance is required for variation to be maintained, for example although the classic 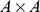 pattern of epistasis (pattern 52, Figure S1) can theoretically avoid fixation when both loci are at allele frequencies of 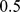 (Figure S2), drift provides sufficient perturbation to prevent it from being maintained at equilibrium (Figure S3).

The consequences of these results are examined below. In summary, we show that a small amount of additive variation is maintained by epistasis but most genetic variation is non-additive; that there is a strong bias in GWA studies that lead to an overestimation of additive effects at QTLs; and that, perhaps counterintuitively, the most powerful way to uncover additive variation under selection is to parameterise the search to include epistatic effects using dense genotype information.

### Most genetic variation maintained under selection is non-additive

**Figure 2:**
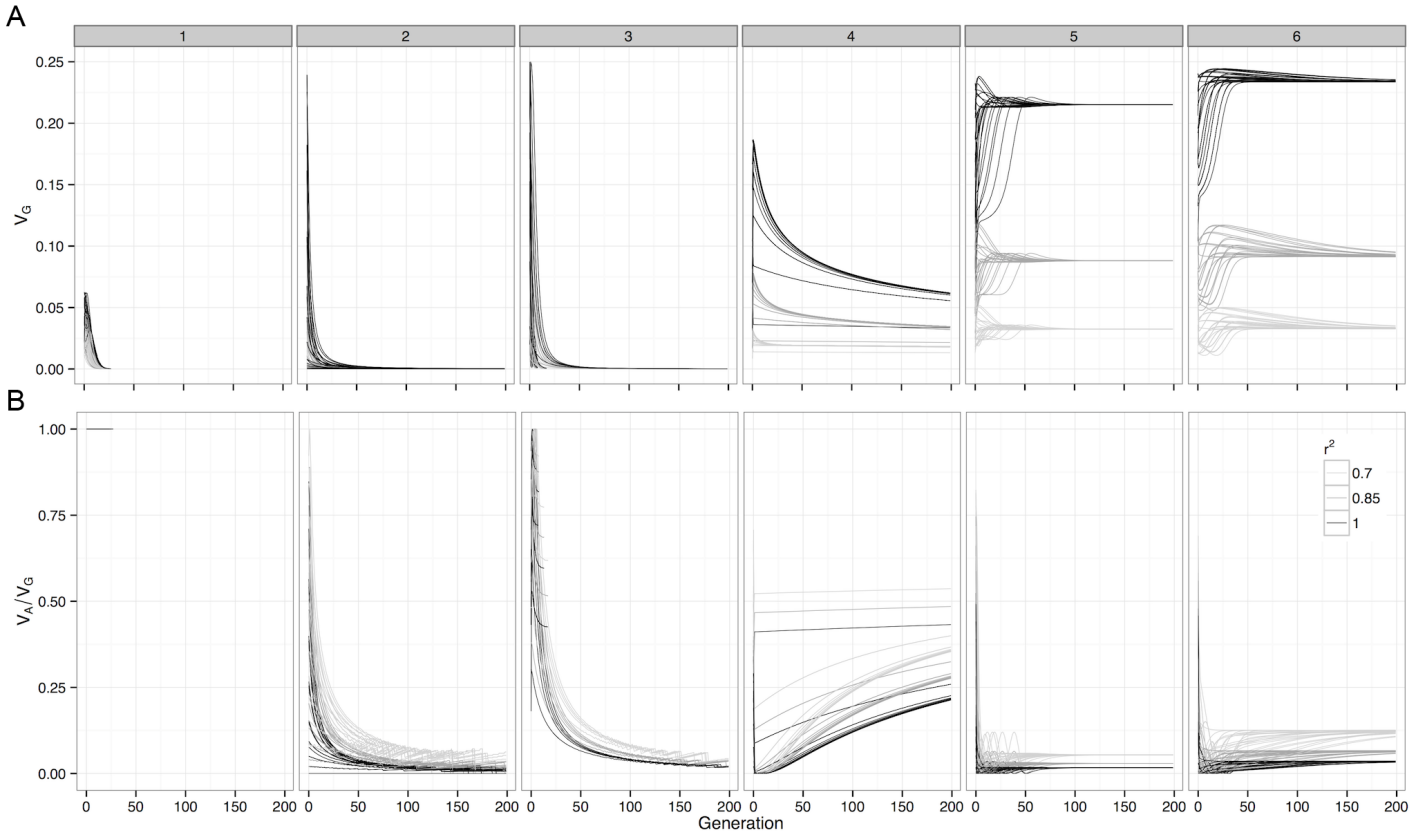
Deterministic change in variance components of G-P maps under selection. For *(a)* Genetic variance and *(b)* Additive variance as a proportion of genetic variation, with initial frequencies of 0.1, 0.3, 0.5, 0.7 and 0.9 enumerated over both loci. The variance decomposition was performed at the causal locus (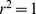), and at SNP pairs that were in incomplete LD with the causal loci. Boxes represent the different G-P maps from Figure 1.

The genetic variance of a G-P map depends on the allele frequencies of the loci involved, and selection drives these allele frequencies to minimise the directional effect of each locus. From this we can calculate the expected changes in genetic variance over time. For many of the patterns of epistasis studied they maintain genetic variance over long evolutionary periods (Figure 2a and Figure S5), as often their allele frequencies can be maintained at intermediate levels. However the majority of this variance is non-additive, with almost all of the additive variance eventually disappearing (Figure 2b and Figure S6).

Although this study assesses a large number of G-P maps, because the parameter space of epistatic G-P maps is effectively infinite the question of how much additive variance can possibly be maintained under selection by a two-locus system still remains. To answer this we used a genetic algorithm to heuristically search the parameter space of the two-locus GP-map, with the objective of finding epistatic patterns that maintain high additive variance over evolutionary time. We should note that the purpose of this exercise is not to identify biologically feasible patterns *per se*, rather it is to assess the propensity for additive variance to be maintained under selection. The epistatic patterns that emerged with the highest level of maintained additive variance, as a proportion of total genetic variance, are shown in Figure 1 (patterns 4–6). The main feature of these patterns is that they exhibit overdominance, and that even in these extreme cases where the algorithm attempts to generate the G-P maps with the largest possible maintained additive variance, it is clear that the majority of genetic variance that is maintained will still be non-additive.

### Incomplete LD leads to the erroneous belief that genetic effects are mostly additive

While it appears that additive variance is difficult to maintain either through independent additive effects or through epistatic interactions, it is clear that most causal effects that have been discovered through GWA studies appear additive in nature [11]. This paradox may result from both ascertainment and confirmation biases that arise when there is incomplete linkage disequilibrium between underlying causal variants and the observed SNPs in a GWA study.

**Figure 3:**
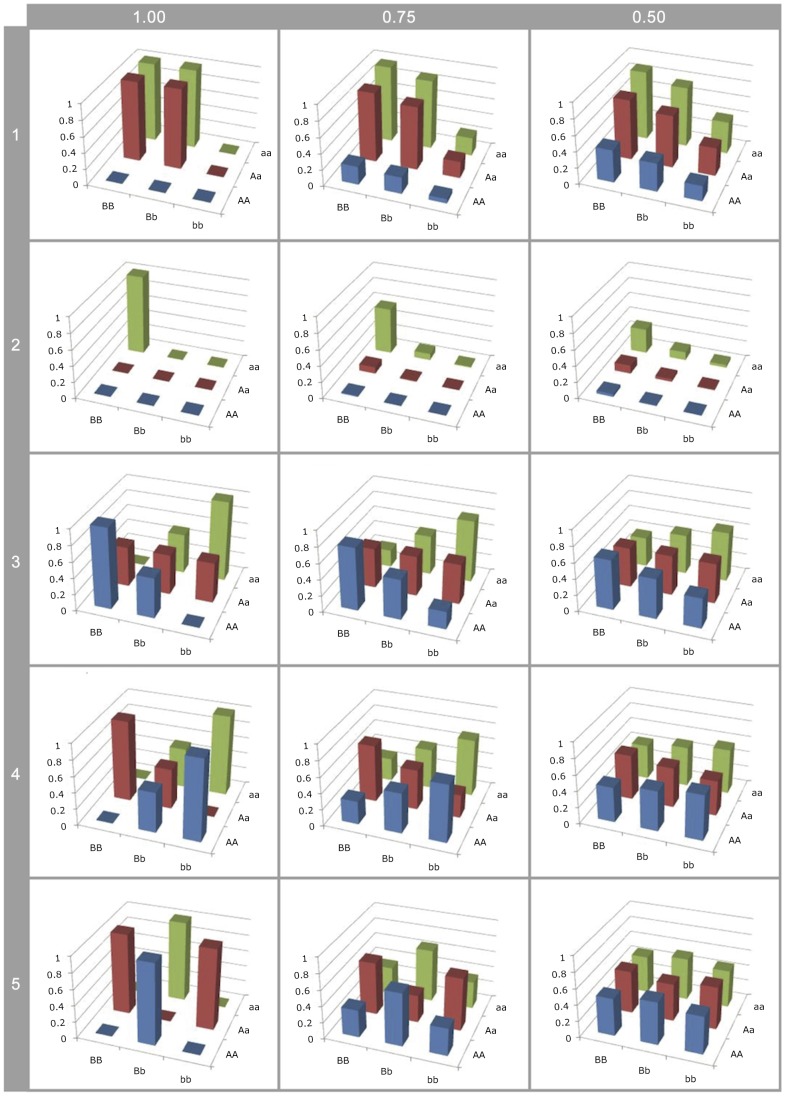
Effect of LD on G-P map estimation. Different G-P maps of causal variants (rows of graphs) deterministically calculated from neighbouring SNPs in different levels of linkage disequilibrium (columns of graphs). All SNP and causal variant frequencies are set to 0.5. Rows 1–2: Canalisation; 3: 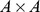; 4: 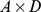; 5: 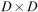.

Figure 2b and Figure S6 show that although estimated genetic variance at observed SNPs decreases as LD with causal variants decreases, the estimated proportion of the variance that is additive actually increases. To illustrate this further Figure 3 shows how estimates of epistatic GP-maps change when LD is reduced, and two important biases can be shown.

Firstly, the higher order variance components (rows 3–5) rapidly haemorrhage genetic variance (see Figure S8), such that even at LD of 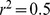 the genotype class means are close to identical. This means that detection is strongly dependent upon high or complete LD, even when effect sizes are large, so most epistatic mutations will remain undetected and their prevalence underestimated. Hence, because additive variance decays linearly with LD [46], at low LD they remain detectable leading to an ascertainment bias for additive vs non-additive effects.

Secondly, with the patterns of canalisation (rows 1–2), as LD reduces although some genetic variance is maintained, the G-P map appears entirely additive. Thus functional maps that confer epistatic effects that can be detected at relatively low LD are likely to be interpreted as being entirely additive. Thus, researchers who attempt to quantify the contribution of non-additive variance from SNPs associated with a trait are liable to incorrectly confirm that most variants act additively.

### Searching for epistatic effects improves power to detect persistent additive variation

Typically GWA studies test each SNP one at a time for additive effects. To explicitly include interaction terms in the search this approach can be extended from one dimension (1D) to two dimensions (2D), where every pair of SNPs is tested jointly for an association [26], [47].

Given that we know the trajectory of allele frequencies under selection, it is possible to ask what the best GWA strategy for detecting evolutionarily likely variants might be. Two main search methods were tested, 1D scans (as are typically performed in GWA studies), and exhaustive 2D scans (previously computationally unfeasible until the availability of more advanced software [47]). For each search method various different model parameterisations were also tested. We used a Bonferroni correction for all methods, so 2D scans had a much more severe multiple testing penalty than 1D scans.

**Figure 4:**
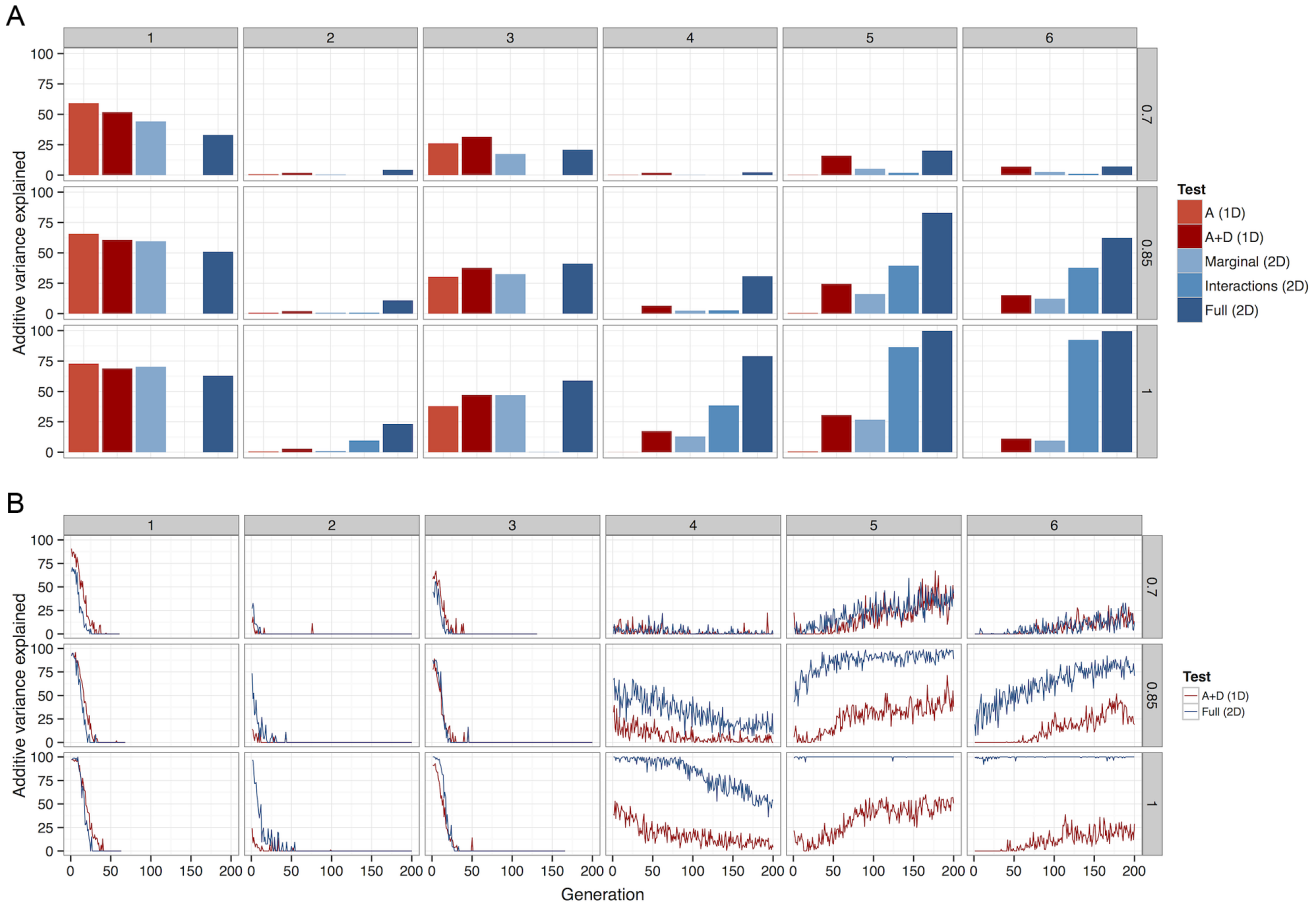
The percentage of total additive variance detected by each of 5 different methods. Columns of graphs refer to G-P maps (Figure 1), rows refer to 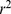 between causal variants and observed SNPs. *(a)* Deterministic calculations were performed 25 times, each with different initial allele frequencies. The percentage of additive variance explained is summed across all runs and generations. *(b)* The summed 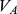 detected at each generation as a percentage of the summed 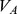 simultaneously present in 50 populations. For clarity, only the most powerful 1D test (A+D) is compared against the most powerful 2D test (full parameterisation). A Bonferroni threshold was used, 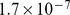 for 1D strategies and 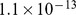 for 2D strategies.

Broadly, the results show two important points. Firstly, there is no single method that is always superior under the conditions that were tested. Secondly, it is very rare that parameterising for additive effects is the most powerful method (Figure 4a and Figure S7). Rather, if LD is no higher than, for example 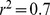, between causal variants and observed SNPs then one dimensional scans, although not particularly powerful in absolute terms, are most effective provided that both additive and dominance effects are modelled (2 d.f. test). In the case of very high LD (*e.g.* dense marker panels, imputed data, sequence data), a strong advantage in power to detect variants at evolutionarily likely frequencies can be conferred by using exhaustive two-dimensional scans and modelling whole genotype effects (8 d.f. test).

A more detailed view of this relationship between LD and detectability is shown in Figure 4b (patterns 4–6). When LD between causal variants and observed SNPs is high, although additive variation exists, much greater power can be achieved in its detection if the search focuses on the larger non-additive variance components. However, as LD decreases, the proportion of the genetic variance that is additive increases, thus one dimensional scans gain an advantage. Nevertheless, as one might expect at low LD there is in general very little power from any method.

It may seem surprising that despite the much larger multiple testing penalty, the 2D scans perform well in terms of power. But there exists a trade-off between the extra variance explained by extending the search into higher dimensions, and the amount of variance required to be detected in order to overcome the multiple testing correction. The results show that because non-additive variance components can be maintained under selection the 2D strategy is conferred an advantage in this trade-off.

## Discussion

The architecture of genetic variation must be understood if we are to make progress in fields such as disease risk prediction, personalised medicine, and animal and crop breeding. This study sought to examine the potential for epistasis to maintain genetic variation under selection, and thus to inform GWA strategies based on these results.

We investigated to what extent deleterious mutations could be maintained as common polymorphisms under selection. A large sample of potential G-P maps were assayed [48] in order to develop a broad picture of the general behaviour of epistasis under selection, and this was extended further by heuristically searching through the parameter space of epistatic G-P maps. It was demonstrated that the maintenance of genetic variation at intermediate frequencies, for traits under selection over evolutionary time, could be achieved through a wide range of two-locus epistatic models. By definition, such is not the case for independent additive effects (Figures S2 and S3). Following on, it was demonstrated that even in the best case scenario, where G-P maps were generated to maximise additive variance, total genetic variance was mostly composed of non-additive components (Figure 2b and Figure S6). This finding is in disagreement with a recent study [28], which showed that for various two-locus epistatic models, the deterministic partitions of genetic variance calculated across different frequency distributions were largely comprised of the additive component. Here we show that those allele frequencies at which additive variance is high (a large proportion of the frequency spectrum), are evolutionarily unstable, thus should epistatic variants be affecting fitness traits then the majority of the variance will be non-additive. Ultimately there is no simple mechanism whereby two-locus epistasis will significantly contribute towards the missing heritability, unless 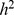 estimates have been contaminated by non-additive genetic components or common environment effects. This is a well-known potential problem with full-sib based estimates and twin studies [30]. Indeed, a recent examination of this problem showed that additive variance estimates could be inflated significantly when complex traits are controlled by epistasis [24].

The results suggest that we should expect significant levels of non-additive variation to be maintained in fitness-related traits. While non-additive variance components are often considered to be nuisance terms in quantitative genetics [49], their existence can be levered to actually improve the detection of additive variance. Here the premise is that if additive variation is observed then there is likely to be an accompanying non-additive genetic component that allows it to persist in the population. Power comparisons were made between 1D and 2D scans, as well as different model parameterisations, with a view to testing the power to detect variants under selection at evolutionarily likely frequencies. Surprisingly, the simplest and most widely used parameterisation, modelling for additive effects in one dimension, was seldom the most powerful approach. On the contrary, because other forms of genetic variance are co-precipitated along with additive variance, by parameterising the tests to include them the power was seen to improve. However, it was observed that even with modest reductions in LD between causal variants and observed SNPs all testing strategies tended to decline in performance rapidly. This leaves researchers in a difficult situation, where the strategy of increasing SNP panel densities as an intuitive response to improve LD coverage comes at a quadratic cost (in the two-locus case) in computation time and multiple testing penalties. An important outcome here is that there is no single test with consistently superior performance, and this resonates with the idea of the “no free lunch” theorem, which states that although competing algorithms will behave differently under different conditions, they will have identical performance when averaged across all conditions [50]. The key in such a situation is to know which conditions are most likely to manifest in the data, and here our argument is that for fitness traits non-additive effects are likely to exist at frequencies where additive variance is minimised.

Although the intention behind the use of the genetic algorithm in this study was to explore the potential for a two-locus system to maintain additive variance, rather than to necessarily identify biologically feasible maps, those maps that emerged did not appear biologically untenable. In fact they can be supported by reports in the literature due to their tendencies for exhibiting heterozygote advantage [51], [52]. The example of the single locus case, overdominance, is central to processes of heterosis and inbreeding depression [52], [53], and has been identified in molecular studies also [54], [55]. Indeed, heterozygote advantage plays an important role in evolutionary theory, as it confers segregational load on a population, and this type of load cannot be purged due to balancing selection, potentially rendering populations susceptible to accumulating a critical mass of such polymorphisms [56]. The idea of a critical mass of deleterious mutations has been widely explored in amictic haploid populations, particularly in the context of Muller's ratchet, and in this case synergistic epistasis has been suggested as a mechanism that could alleviate the problem in some situations [57], [58]. This study may offer a similar answer for the analogous problem of segregational load in diploid populations, because it can be observed that while patterns of overdominance (Figure S3, pattern 55) form a stable equilibrium, small perturbations to this G-P map through the introduction of an interacting locus (*e.g.* patterns 45, 47, 53) could destabilise the equilibrium and lead to eventual fixation.

It is important to note that the processes underlying stasis and missing heritability are unlikely to be caused by any single factor. For example, a compelling argument is that though most traits exhibit genetic variation, selection acts upon multidimensional trait space in which there is no genetic variation [59], and this will hold under an additive model of genetic variation. It is also important to consider the manner in which traits of interests, such as human diseases, are involved in fitness. For example in an assessment of selection signatures on SNPs implicated in type 1 diabetes it has been shown that the causal alleles have undergone positive selection to a greater extent than protective alleles, while with Crohn's disease the converse is true [60]. In the case of both diseases more variants are being discovered as sample sizes increase [61], [62], but given that only a small proportion of the total heritability has so far been explained, and the search has concentrated on additive variants only, inferences about the genetic architecture cannot be made.

Occam's razor can be invoked to justify the additive paradigm used in GWA studies [28]. But the analyses presented here demonstrate that perhaps rejecting more complex models in favour of simple ones should not always be the automatic choice. With sample sizes growing and with the tools now available to search for epistasis in a computationally efficient manner (*e.g.* [47], [63]–[66]) it should be possible to explore the genetic architecture of complex traits in directions that were not previously possible.

## Methods

We were interested in simulating the behaviour of epistatic interactions under selection in order to ask the questions i) for how long and at which frequencies are deleterious mutations maintained under selection, ii) how much additive variance can this produce, and iii) what is the best strategy to identify evolutionarily persistent variation. We approached this problem through two methods, using stochastic simulations and by calculating the expected trajectories deterministically, and overall a range of different epistatic and dominant G-P maps were assessed. In addition, we heuristically searched the two-locus genotype-phenotype parameter space using a genetic algorithm to assess what the upper limit on how much additive genetic variation can be maintained under selection.

### Deterministic simulations

The evolutionary fate of an arbitrary two-locus epistatic fitness pattern can be characterised by the allele frequencies and recombination fraction of the two loci as a Markovian process. Therefore it is straightforward to calculate the trajectory of allele frequencies over evolutionary time for a wide range of epistatic patterns. For each G-P map, deterministic simulations were performed with varying conditions for initial allele frequencies (25 initial allele frequencies enumerating the set 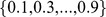 over both loci) and linkage disequilibrium between the linked and causal SNPs (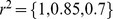). Variance components and expected test statistics for different parameterisations and under different assumed search strategies were calculated.

#### Two-locus frequency calculations

For a two-locus gametic fitness pattern 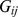, where each value of 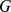 represents the mean phenotypic value for individuals with haplotypes 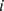 and 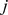,
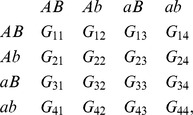
assuming that 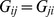, this can be related to the two-locus G-P map  as:
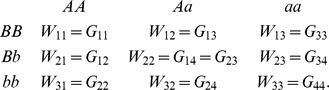
We can calculate the expected haplotype frequencies 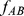, 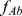, 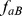, 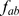 after one generation based on selection using [45] and [67]. Here, the haplotype frequencies of the current generation are represented as 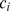 where 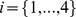 denotes each haplotype in the order listed above, and 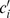 is the haplotype frequency of the next generation:
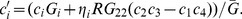
(1)Here
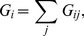
(2)

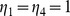
, 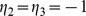, 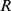 is the recombination fraction between the two loci (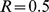 denotes the two loci are effectively on separate chromosomes) and
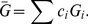
(3)We ran the simulations for 200 generations, or until one of the loci becomes fixed. If the minor allele from at least one locus 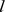 breaks the condition
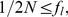
(4)where 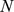 is the population size (arbitrarily set to 1000 for these simulations), the epistatic pattern is considered fixed. While this condition is satisfied, expected variance decomposition and statistical power are assessed on the system at each generation.

#### Variance decomposition

As the allele frequencies change due to selection, while the functional epistatic pattern remains the same the variance components are liable to change. The following calculations, taken from [68], can be used to calculate the marginal additive variances at each locus in a pairwise epistatic interaction for populations at each generation of the simulations. Given marginal fitnesses at the three genotypes at locus A
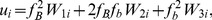
(5)and at locus B
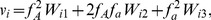
(6)the marginal additive variance at locus A is
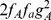
(7)and the marginal additive variance at locus B is
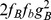
(8)where
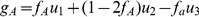
(9)and
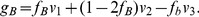
(10)However, because linkage disequilibrium can be generated between interacting loci under selection (Figure S4) it is incorrect to quantify the additive variance as the sum of the two marginal variances. Instead, we use the decomposition method detailed in [69] and [70] to calculate the total additive genetic variance in a two-locus system as
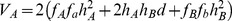
(11)where
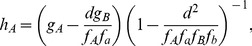
(12)

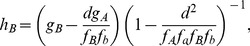
(13)and
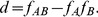
(14)


It should be noted that these calculations assume Hardy-Weinburg equilibrium, and selection is likely to generate pseudo LD between unlinked markers, as well as favour certain genotypes over others which results in a violation of this assumption. However there is currently no known two-locus variance decomposition method that maintains orthogonality when the two loci are under linkage disequilibrium and Hardy-Weinburg disequilibrium [71], therefore correct estimates of variance components often cannot be made. However, given that current testing strategies still use the incomplete extant methods, we can examine their behaviour without the requirement of orthogonality between the non-additive components. We use the NOIA method of decomposition [71] to calculate total genetic variance (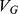) and the 8 variance components, 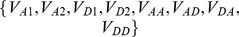.

#### Detection of additive variance

By specifying the broad-sense heritability 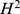 of a fitness trait at generation 0 for each simulation it is possible to calculate expected F-test performances under different parameterisations and scan strategies. During the simulation selection can modify 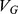 by changing allele frequencies, but the non-genetic variance, 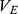, remains constant as a function of 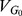, the genetic variance at initial allele frequencies:
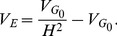
(15)


We wanted to find, given a GWAS testing strategy wherein a SNP's contribution to the narrow-sense heritability is only considered if the test statistic meets some significance threshold, how best to parameterise the hypothesis tests to maximise the expected amount of additive variance significantly identified for any given simulation time point. Using an F-test,
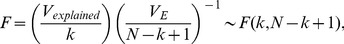
(16)where 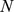 is the sample size and 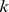 is the number of parameters in the model, we compared different parameterisations of 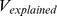 for exhaustive one and two dimensional scans by quantifying how much of the total additive variance in the two-locus system was detected using different GWAS strategies.

For the one dimensional strategy tests for purely additive effects (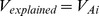; 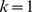) or complete marginal effects (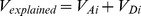; 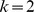) were performed at each locus 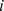. A significance threshold of 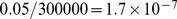 was set. If met at only one locus 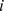 then 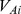 additive variance was considered detected. If met at both loci then the total additive variance 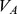 was considered detected.

For the two dimensional strategy three different parameterisations were compared under the conditions of an exhaustive two dimensional scan. These were for purely marginal effects across both loci (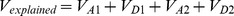; 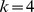), purely epistatic effects (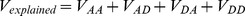; 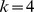), and for total genetic variance (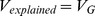; 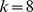). The significance threshold was set at 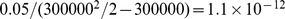. If the pairwise test met this threshold then, for the purposes of understanding the efficacy of two dimensional strategies at detecting narrow sense heritability, the total additive variance 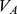 across both loci was deemed to have been detected.

#### Incomplete LD between causal variants and observed SNPs

We considered how variance decomposition and testing strategies were affected when the observed SNPs were at different levels of linkage disequilibrium with the causal variants (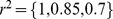). To do this, we transformed the G-P map of the causal loci (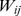), to the G-P map of the observed SNPs that have some level of LD with the causal loci (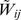). This is constructed by considering that when LD is reduced, the genotype class means of 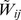 are a composite of not only 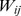, but also other genotype class means, and the expected contribution of the other class means depends on the level of recombination between the observed SNPs and the causal variants. We performed the above variance decomposition calculations on 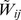, assuming that
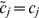
(17)where 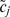 are the gametic frequencies of the observed SNPs. For simplicity, only the causal variants were inherited from one generation to the next, with new linked SNPs being composed at each new generation. The G-P map 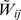 is calculated as
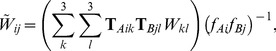
(18)where the frequencies 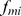 are the expected genotype frequencies for the 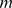 interacting causal variants 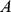 and 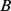, such that
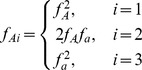
(19)and
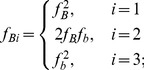
(20)and matrix 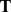 is 3-dimensional with dimensions 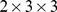, where 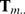 is defined as
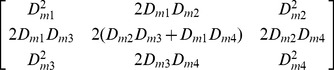
(21)where the four gametic frequencies (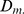) for the interacting causal loci (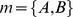), and their correlated observed SNPs were calculated as:
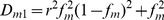
(22)

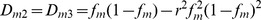
(23)

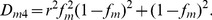
(24)


### Genetic algorithm for generating epistatic patterns

The purpose of genetic algorithms is to heuristically search a large solution domain for optimal model parameters whilst avoiding a computationally prohibitive exhaustive search [72]. In this case, the algorithm is used to search for two-locus epistatic fitness patterns 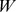 that simultaneously maximise additive genetic variance and avoid fixation through selection, where 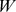 is a 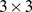 G-P map whose values represent the fitness associated with each two-locus genotype. In this case the building blocks are the nine two-locus genotype class means that comprise the G-P map.

#### Initialisation

Initially a set 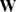 of 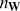 randomly generated candidate patterns 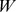 are created by sampling values for each of the 9 cells from a uniform distribution, and then scaling all values so that the maximum and minimum values for each 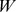 are 1 and 0 respectively.

#### Selection

The candidate patterns are assessed based on two rounds of selection: expected time to fixation and expected level of additive variance generated. A set 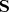 of simulations are initialised given sets of 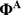 and 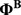 initial allele frequencies for loci 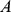 and 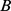 respectively, such that 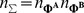 (*e.g.* 25 simulations initialised by enumerating all combinations of the sets 
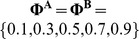
 across two loci). Allele frequency changes and fixation are measured as in equations (1) and (4) respectively at each generation 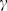 for 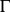 generations. For the candidate pattern to be considered for selection at least 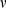 of its simulations must remain unfixed after 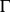 generations. For each candidate pattern its total additive genetic variance 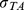 over time is calculated by summing the joint additive variance for both loci (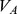) for each generation starting after generation 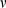 and until either fixation or generation 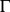, and across all initial allele frequency conditions:
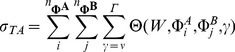
(25)where 
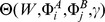
 is the additive variance at generation 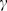 of simulation 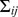. The 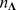 candidate patterns with the largest total additive variances are selected for the next round, comprising the set 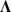, or if no candidate patterns reach the threshold 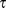 then all patterns are randomly initialised again.

#### Reproduction

The set of 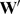 candidate patterns for the next round of selection is comprised of the 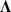 patterns selected from the current round, a set of 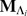 mutations for each selected pattern 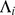, and a set of 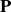 random patterns produced as in the initialisation step, thus 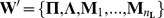. Mutation is performed by adjusting each element of the candidate pattern:
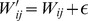
(26)where
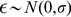
(27)and then scaling to the boundaries 0 and 1 as in the initialisation step.

#### Termination

The algorithm is performed for 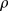 rounds. Because the set 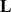 candidate patterns from the previous round are always included in the following round, the maximum score will never decrease. Therefore the optimal epistatic pattern is the considered to be the highest scoring candidate pattern in the final round. Different patterns can be generated by rerunning the entire process with different random seeds.

The code for this algorithm is available at https://github.com/explodecomputer/epiSpaces/.

### Population simulations

To consider the potential impact of genetic drift and random noise on the conclusions from the deterministic simulations, similar conditions were recreated heuristically on randomly generated populations. For each epistatic pattern we generated 300 populations of 1000 individuals. Each individual has a two-locus genotype 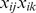 and a corresponding phenotype 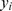 such that
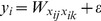
(28)where
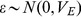
(29)and 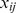 and 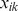 were the fitness values for indvidual 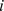 corresponding to the G-P map 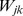. The non-genetic variance of the trait was defined at generation 0 as in equation 15 and remained constant at each generation. The heritability, 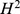, was set to 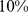 at generation 0. Each generation 500 individuals were sampled from a discrete probability distribution where the individual's phenotype was the relative probability of being sampled, and from these 250 random pairings were made to produce 1000 offspring for the next generation. Phenotypes for each new individual were created at each generation as in equation 28, and simulations continued until at least one locus reached fixation. The initial allele frequencies were 0.5 for each locus, and the simulations ran for 200 generations or until at least one locus became fixed.

The code for this algorithm is available at https://github.com/explodecomputer/epiFit/.

## Supporting Information

Figure S1 G-P maps. *1* Neutral; *2–51* Enumeration of all binary trait patterns, excluding reflections, rotations and inversions, as derived by [48] (6 and 29 are non-episatatic); *52–56* Additive×Additive, Additive×Dominance, Dominance×Dominance, Over-dominance, additive.
(TIF)

Figure S2 Deterministic trajectory of allele frequencies as in Figure 1 (row 2), but for an extended set of patterns (detailed in Figure S1)
(TIF)

Figure S3 Simulated trajectory of allele frequencies as in Figure 1 (row 3), but for an extended set of patterns (detailed in Figure S1)
(TIF)

Figure S4 Quasi-LD generated by selection. For the 25 deterministic simulations the expected quasi-LD between the physically unlinked causal SNPs was calculated. It can be seen that significant levels are generated, such that orthogonal standard parameterisation methods would violate assumptions of independence. Boxes represent different G-P maps from Figure S1.
(TIF)

Figure S5 Deterministic change in genetic variance for loci under selection exhibiting various epistatic patterns (Figure S1), when LD between the causal variants and observed SNPs varies. For clarity, only the results from initial frequencies of 0.5 at both loci are shown. Boxes represent different G-P maps from Figure S1.
(TIF)

Figure S6 As in Figure S5, but this time showing the proportion of the genetic variance that is additive.
(TIF)

Figure S7As in Figure 4a, but for only three tests - Additive in one dimension (A (1D)), genotype in one dimension (A+D (1D)), and full epistatic in two dimensions (F (2D)). Each box has the additive variance detected across all populations and generations as a proportion of the total additive variance that was created for each test when the observed SNPs were in varying levels of LD with the causal variants. For 44 patterns the full epistatic test is most powerful when 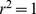, but when 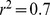 it is never the most powerful, rather 39 patterns are best detected by the one dimensional genotype parameterisation.(TIF)

Figure S8Relationship between genetic variance of observed SNPs (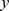 axis) and their linkage disequilibrium with causal variants (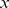 axis). Observed SNPs have the same allele frequency as their linked causal variants, and there is no linkage disequilibrium between causal variants or between observed SNPs. The blue line represents a purely additive G-P map, faint black lines each represent the 55 dominant or epistatic G-P maps in Figure S1, and the black dashed line represents the smoothed average of all black lines. Allele frequencies of G-P maps are represented by boxes, the frequency of locus A horizontally and locus B vertically.(TIF)
