# Supplementary figures and images for: An Evolutionary Perspective on Epistasis and the Missing Heritability
Source: PLoS Genet. 2013 Feb 28;9(2):e1003295. doi: 10.1371/journal.pgen.1003295 (PMC3585114; doi:10.1371/journal.pgen.1003295)

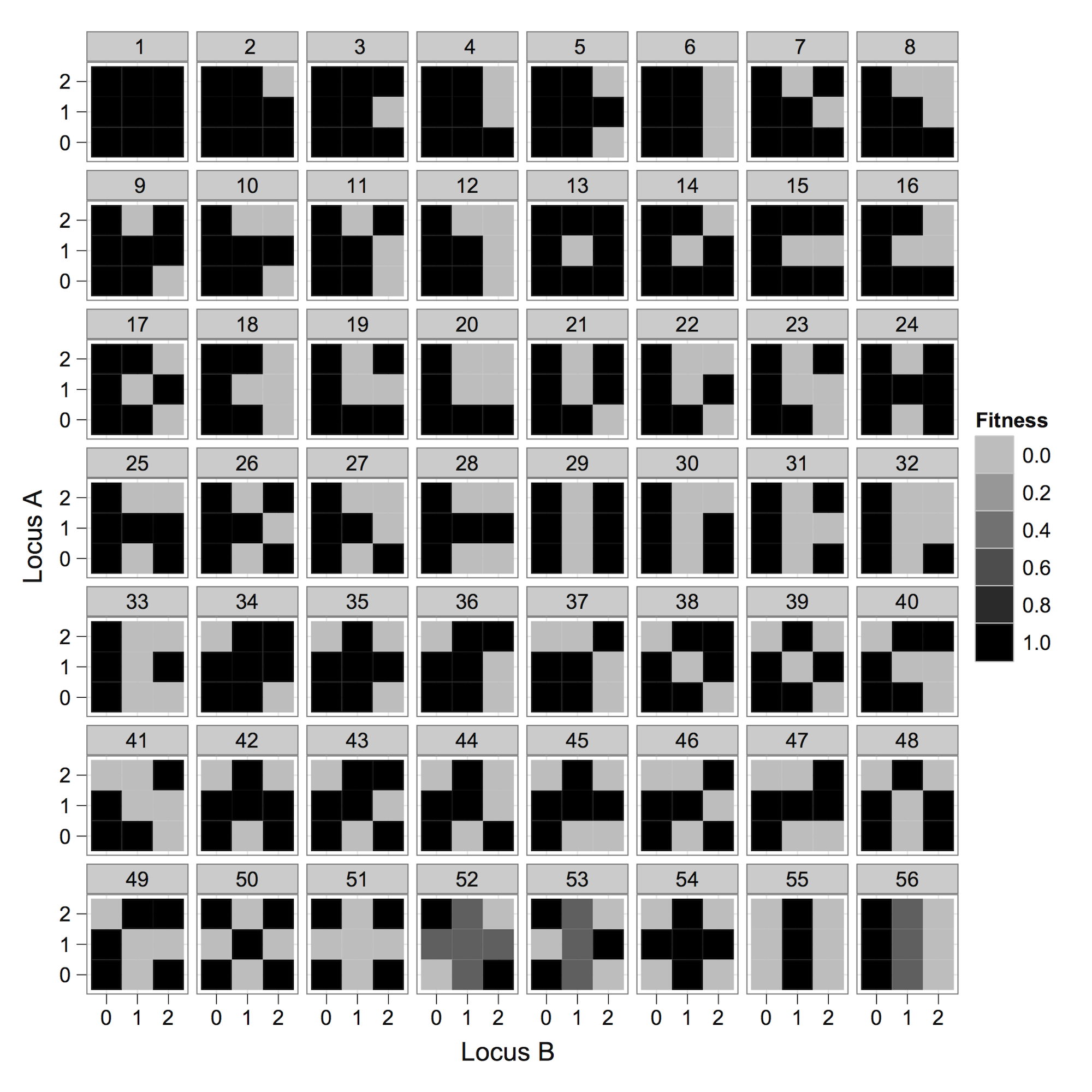

Supplement: Figure S1 — G-P maps. 1 Neutral; 2–51 Enumeration of all binary trait patterns, excluding reflections, rotations and inversions, as derived by [48] (6 and 29 are non-episatatic); 52–56 Additive×Additive, Additive×Dominance, Dominance×Dominance, Over-dominance, additive. (TIF) [file pgen.1003295.s001.tif]

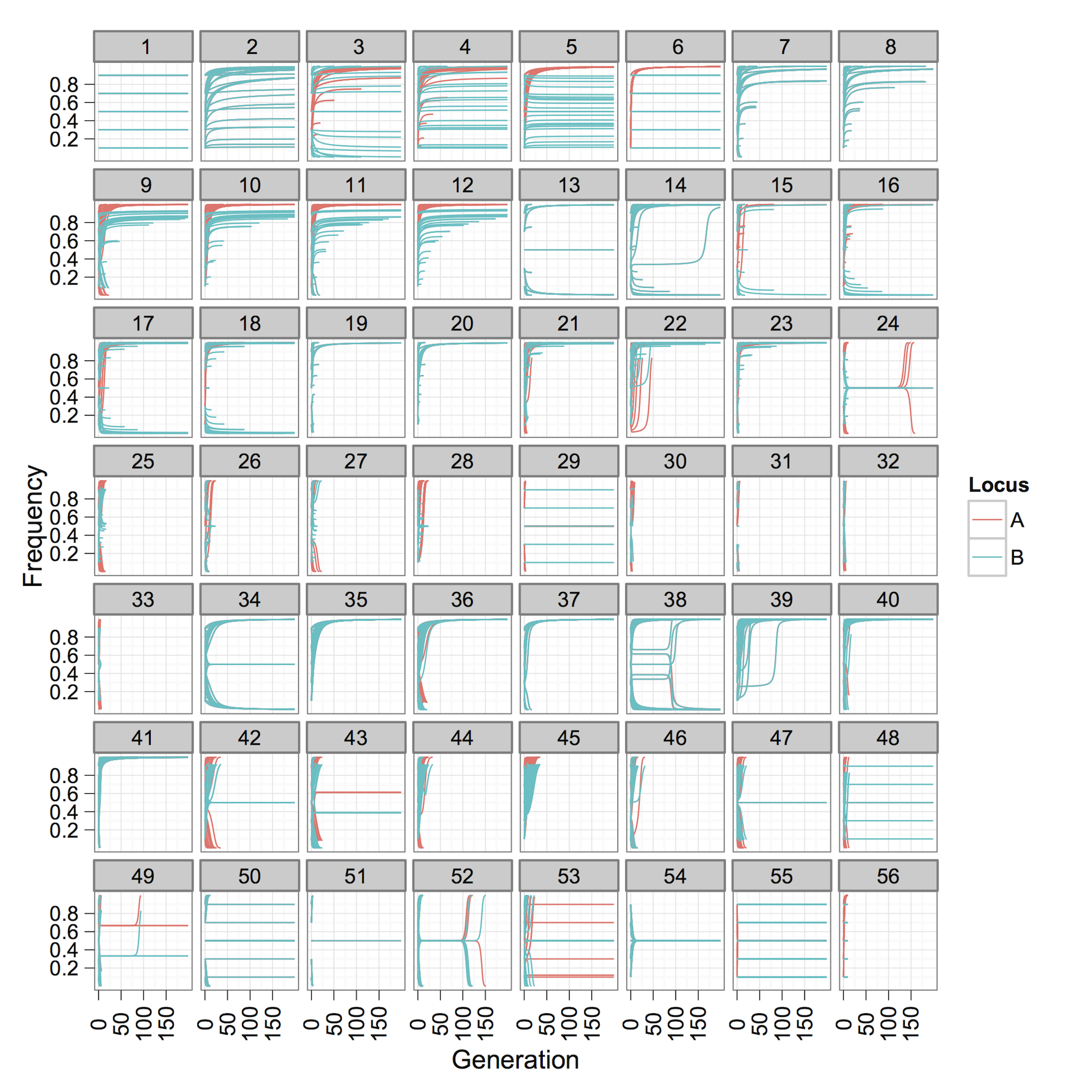

Supplement: Figure S2 — Deterministic trajectory of allele frequencies as in Figure 1 (row 2), but for an extended set of patterns (detailed in Figure S1) (TIF) [file pgen.1003295.s002.tif]

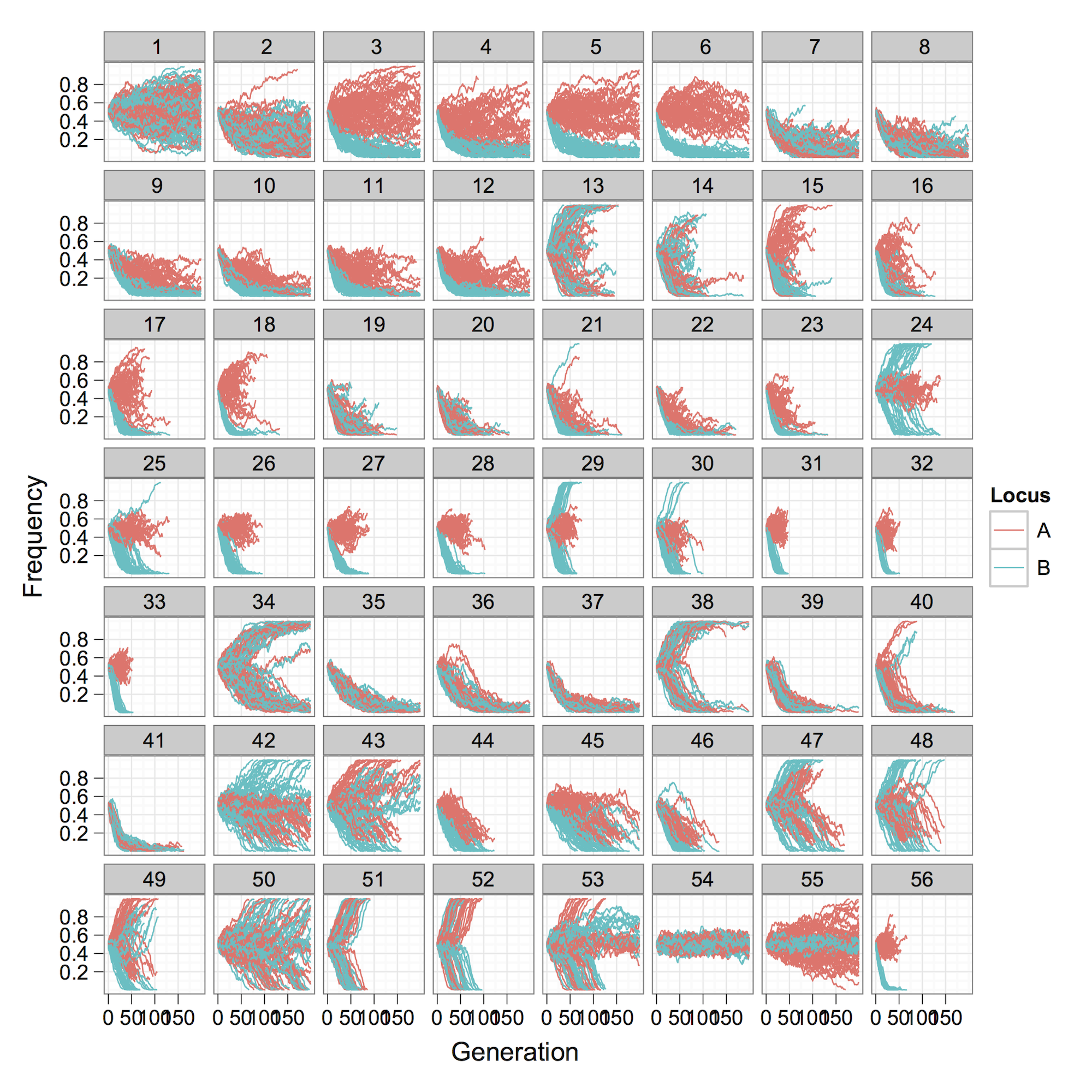

Supplement: Figure S3 — Simulated trajectory of allele frequencies as in Figure 1 (row 3), but for an extended set of patterns (detailed in Figure S1) (TIF) [file pgen.1003295.s003.tif]

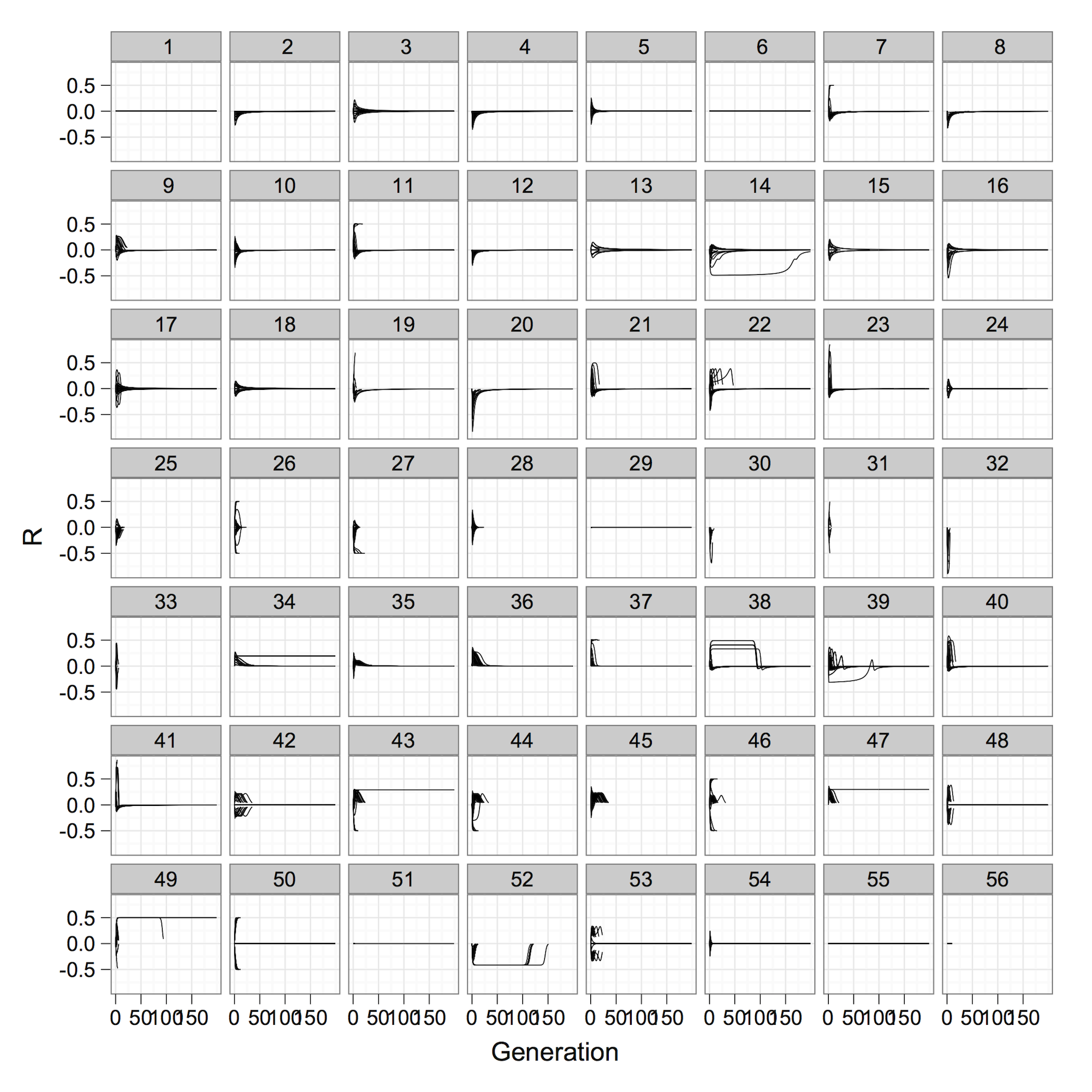

Supplement: Figure S4 — Quasi-LD generated by selection. For the 25 deterministic simulations the expected quasi-LD between the physically unlinked causal SNPs was calculated. It can be seen that significant levels are generated, such that orthogonal standard parameterisation methods would violate assumptions of independence. Boxes represent different G-P maps from Figure S1. (TIF) [file pgen.1003295.s004.tif]

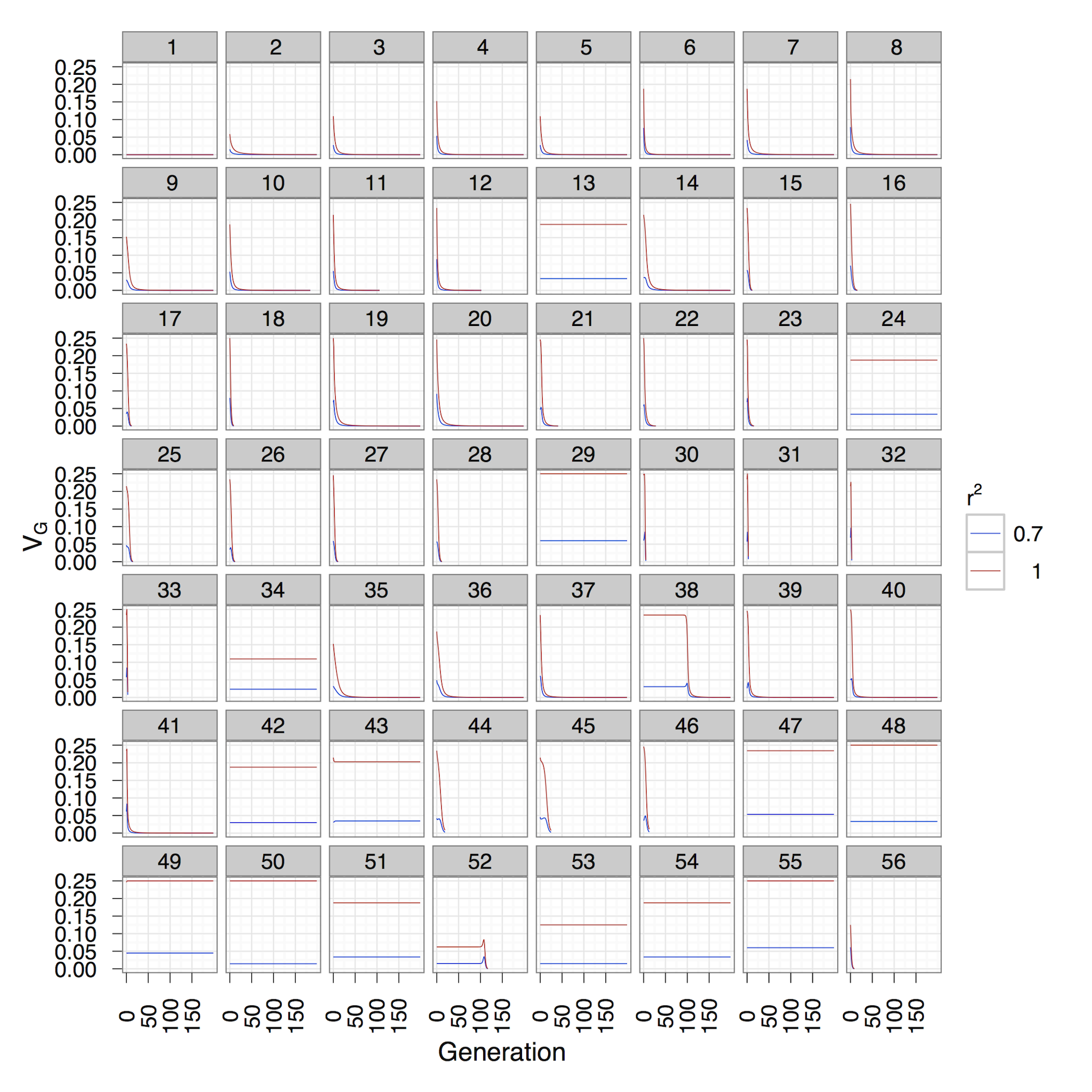

Supplement: Figure S5 — Deterministic change in genetic variance for loci under selection exhibiting various epistatic patterns (Figure S1), when LD between the causal variants and observed SNPs varies. For clarity, only the results from initial frequencies of 0.5 at both loci are shown. Boxes represent different G-P maps from Figure S1. (TIF) [file pgen.1003295.s005.tif]

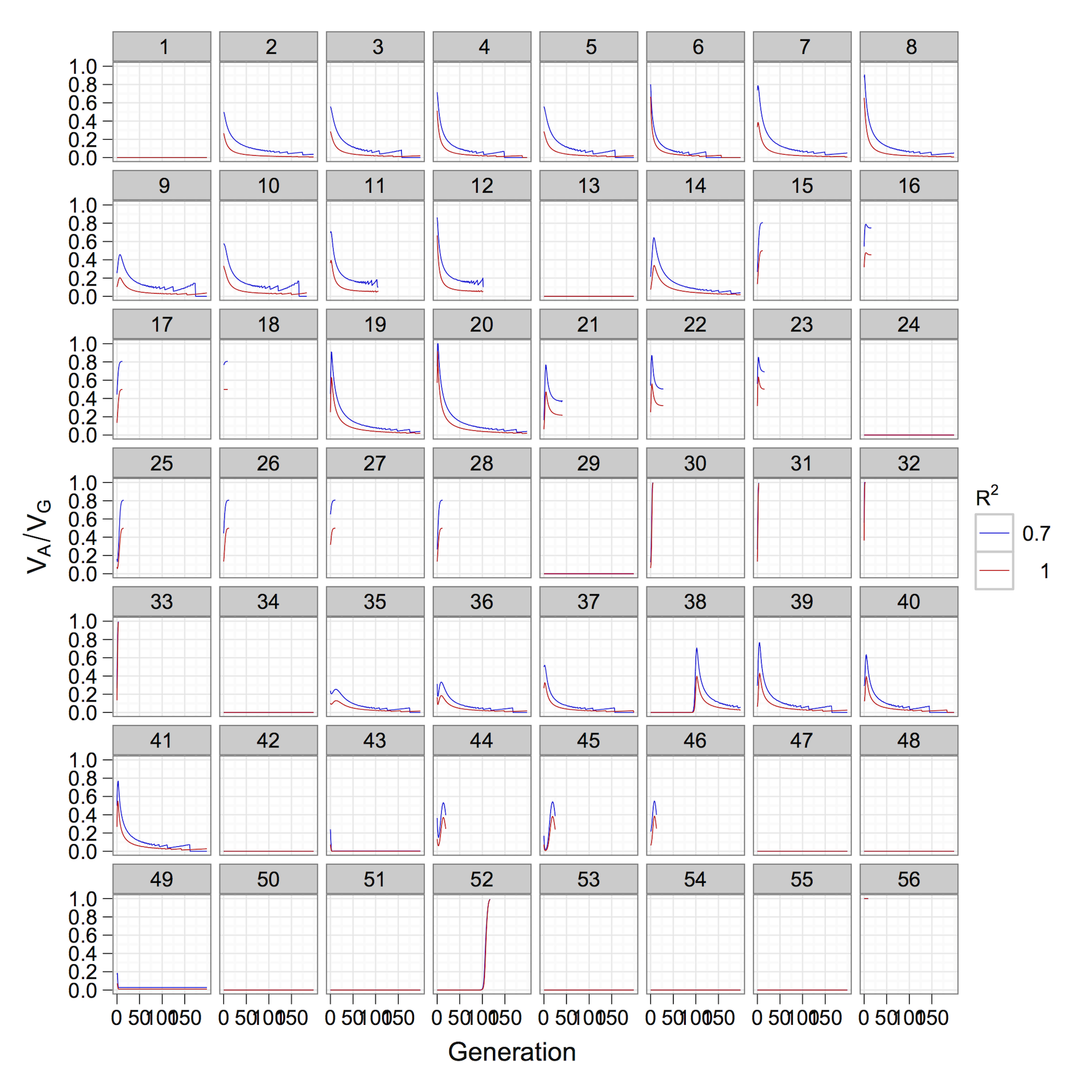

Supplement: Figure S6 — As in Figure S5, but this time showing the proportion of the genetic variance that is additive. (TIF) [file pgen.1003295.s006.tif]

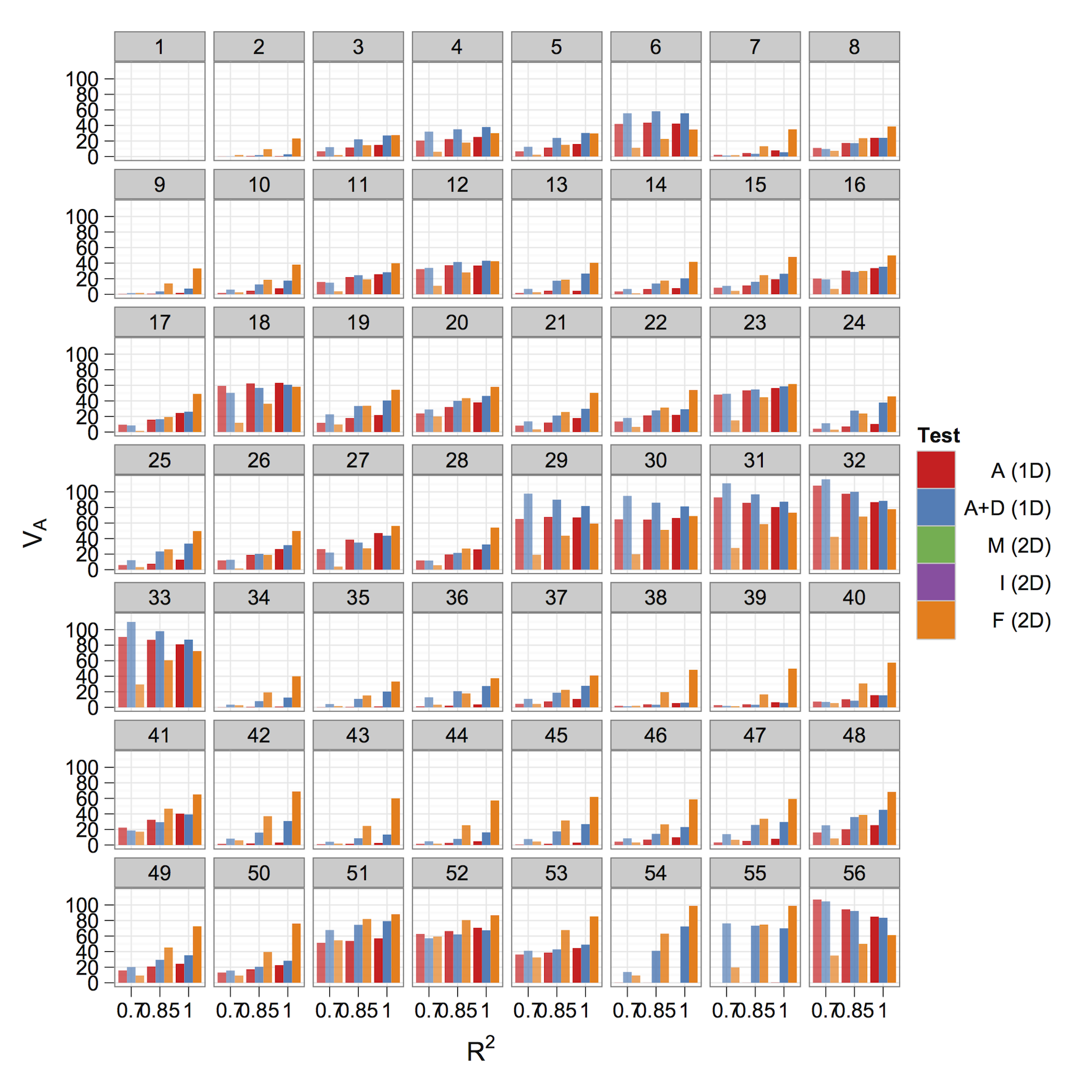

Supplement: Figure S7 — As in Figure 4a, but for only three tests - Additive in one dimension (A (1D)), genotype in one dimension (A+D (1D)), and full epistatic in two dimensions (F (2D)). Each box has the additive variance detected across all populations and generations as a proportion of the total additive variance that was created for each test when the observed SNPs were in varying levels of LD with the causal variants. For 44 patterns the full epistatic test is most powerful when , but when it is never the most powerful, rather 39 patterns are best detected by the one dimensional genotype parameterisation. (TIF) [file pgen.1003295.s007.tif]

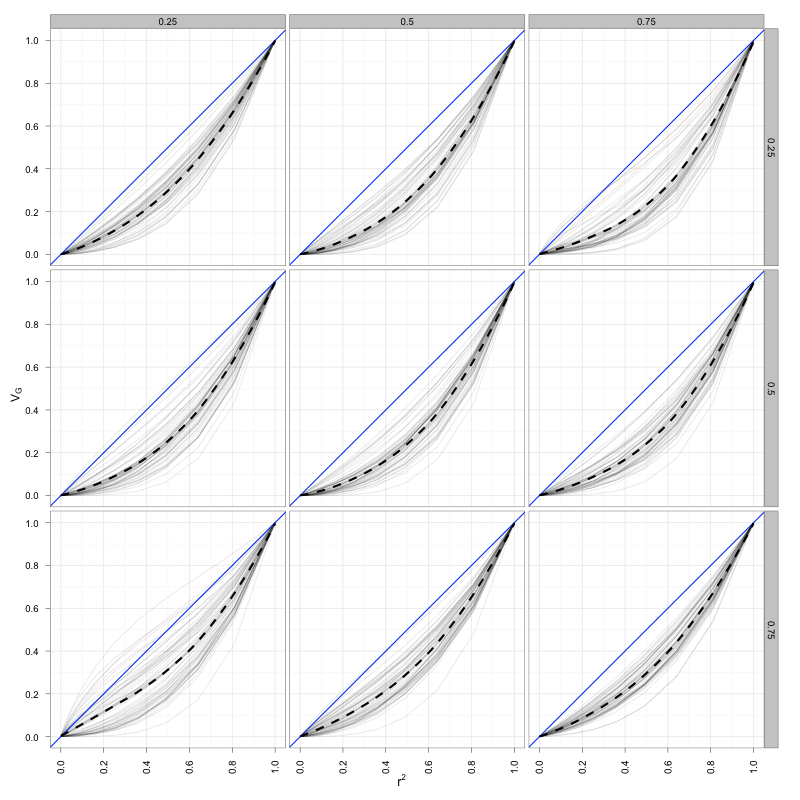

Supplement: Figure S8 — Relationship between genetic variance of observed SNPs ( axis) and their linkage disequilibrium with causal variants ( axis). Observed SNPs have the same allele frequency as their linked causal variants, and there is no linkage disequilibrium between causal variants or between observed SNPs. The blue line represents a purely additive G-P map, faint black lines each represent the 55 dominant or epistatic G-P maps in Figure S1, and the black dashed line represents the smoothed average of all black lines. Allele frequencies of G-P maps are represented by boxes, the frequency of locus A horizontally and locus B vertically. (TIF) [file pgen.1003295.s008.tif]
